# CircGLIS3 Promotes High-Grade Glioma Invasion via Modulating Ezrin Phosphorylation

**DOI:** 10.3389/fcell.2021.663207

**Published:** 2021-09-03

**Authors:** Yan Li, Jiansheng Chen, Zetao Chen, Xiangdong Xu, Jun Weng, Yuxuan Zhang, Yunzhao Mo, Yang Liu, Jihui Wang, Yiquan Ke

**Affiliations:** ^1^The National Key Clinical Specialty, The Engineering Technology Research Center of Education Ministry of China, Guangdong Provincial Key Laboratory on Brain Function Repair and Regeneration, Department of Neurosurgery, Zhujiang Hospital, Southern Medical University, Guangzhou, China; ^2^Department of Neurosurgery, Huizhou Municipal Central Hospital, Huizhou, China; ^3^Department of Hepticbile Surgery, Zhujiang Hospital, Southern Medical University, Guangzhou, China

**Keywords:** circular RNA, Ezrin, high-grade glioma, invasion, protein binding

## Abstract

High-grade glioma is highly invasive and malignant, resistant to combined therapies, and easy to relapse. A better understanding of circular RNA (circRNA) biological function in high-grade glioma might contribute to the therapeutic efficacy. Here, a circRNA merely upregulated in high-grade glioma, circGLIS3 (hsa_circ_0002874, originating from exon 2 of *GLIS3*), was validated by microarray and Real-time quantitative reverse transcription PCR (qRT-PCR). The role of circGLIS3 in glioma was assessed by functional experiments both *in vitro* and *in vivo*. Fluorescence *in situ* hybridization (FISH), RNA pull-down, RNA immunoprecipitation (RIP), and immunohistochemical staining were performed for mechanistic study. Cocultured brain endothelial cells with glioma explored the role of exosome-derived circGLIS3 in the glioma microenvironment. We found that upregulation of circGLIS3 promoted glioma cell migration and invasion and showed aggressive characteristics in tumor-bearing mice. Mechanistically, we found that circGLIS3 could promote the Ezrin T567 phosphorylation level. Moreover, circGLIS3 could be excreted by glioma through exosomes and induced endothelial cell angiogenesis. Our findings indicate that circGLIS3 is upregulated in high-grade glioma and contributes to the invasion and angiogenesis of glioma via modulating Ezrin T567 phosphorylation.

## Introduction

Gliomas account for 25.1% of all primary brain and other central nervous system tumors according to CBTRUS (Central Brain Tumor Registry of the United States); approximately 70% of gliomas are high-grade gliomas (HGG, WHO grade III, and WHO grade IV gliomas) (Ostrom et al., 2020). HGG are highly aggressive and lethal malignancies, especially glioblastoma multiforme, with a 5-year relative survival rate of 7.2% (Ostrom et al., 2020). HGG are characterized by wide invasion; they can invade through vascular and brain parenchyma, which shelters them from surgery and radiotherapy and causes tumor recurrence in a short time (Cuddapah et al., 2014). Meanwhile, HGG have abundant and aberrant vasculature; they advance in tumor progress through multiple angiogenesis mechanisms (Kane, 2019). Although comprehensive treatment of surgery combined with chemoradiotherapy provides a benefit for patients with HGG, it remains largely refractory to treatment due to the high invasiveness and angiogenesis characters (Marenco-Hillembrand et al., 2020). Despite that developing treatments on reducing glioma progress such as anti-vascularization drugs are under investigation (Kim et al., 2018), the prognosis of HGG is still badly poor. It is necessary to explore the underlying mechanisms of glioma etiology for advancing the development of new effective therapy.

Circular RNA (circRNA) is a novel class of RNA firstly found in 1976 (Sanger et al., 1976). With the development of deep sequencing and bioinformation, a growing evidence showed that circRNA can widely participate in multiple physiological and pathological processes, including tumor oncology (Hansen et al., 2013; Chen et al., 2019; Huang et al., 2019). In addition, circRNA is abundant in mammalian brain and found dysregulated in glioma, indicating various regulatory functions of circRNA in glioma (Rybak-Wolf et al., 2015; Song et al., 2016; Sun et al., 2020). Widely proposed mechanisms of circRNA include sequestrating microRNA to restore mRNA from degradation (Zheng et al., 2020), binding with proteins (usually called RNA-binding protein, RBP) (Zang et al., 2020), and competing with its linear homologous counterparts (Ashwal-Fluss et al., 2014). CircRNA can also be translated into short functional peptides (Yang et al., 2018). However, studies on how circRNA interacts with proteins in glioma are rare. He et al. (2019) found that the RBP FUS regulates the expression of circ_002136 upstream to promote glioma angiogenesis. Only recently, Lou et al. (2020) discovered CDR1 as directly binding to the p53 DBD domain and protecting it from MDM2 ubiquitination. Above all, further investigations are required to fully understand the function and underlying mechanism of circRNA in glioma.

Considering the gap in circRNA research, we performed circRNA microarray analysis and focused on circGLIS3 (circBase ID: hsa_circ_0002874), which is upregulated in HGG. We proved that circGLIS3 can promote glioma invasion both *in vitro* and *in vivo*. Mechanistically, we confirmed that circGLIS3 binds with p-Ezrin(T567) in glioma. Ezrin comes from the ezrin/radixin/moesin (ERM) family, which is crucial for cell membrane structure and signal transduction (McClatchey, 2014; Zhang et al., 2020). Aberrant phosphorylation of ezrin plays a part in metastasis of many tumors (Zhan et al., 2019). Moreover, we found that circGLIS3 can be secreted by glioma through exosomes and promote human brain microvascular endothelial cells (hBMEC) vascularization. In conclusion, our study reveals that circGLIS3 promotes high-grade glioma invasion and angiogenesis by modulating Ezrin T567 phosphorylation.

## Materials and Methods

### Human Glioma and Non-tumor Brain Tissue Samples

We collected 28 glioma samples and 5 non-tumor brain tissues (NBT) from patients without glioma who were required for intracranial decompression surgery for other reasons (Supplementary Table 1). All patients enrolled in this study signed an informed consent. The study methodologies conformed to the standards set by the Declaration of Helsinki. All samples were separated and stored in 30 min after surgical resection, for microarray, RT-PCR, and isolation of primary glioma cells. All studies were approved by the Clinical Research Ethics Committee of Zhujiang Hospital, Southern Medical University. All patients signed consent forms.

### Circular RNA Microarray

Total RNAs extracted from the 16 NBT and glioma samples of different grades were cryo-pulverized using BioPulverizer (BioSpec) and homogenized with TRIzol (Invitrogen Life Technologies) by Mini-BeadBeater-16 (BioSpec). RNA was collected from each sample based on Arraystar’s standard protocols. Briefly, total RNAs were digested with RNase R (Epicentre, Inc.) to remove linear RNAs and enrich circRNAs. The enriched circRNAs were amplified and transcribed into Cy3 fluorescent cRNA utilizing a random priming method (Arraystar Super RNA Labeling Kit; Arraystar). The labeled cRNAs were hybridized onto Arraystar Human circRNA Array v2 (8 × 15K, Arraystar). After having washed the slides, the arrays were scanned using the Agilent Scanner G2505C. Scanned images were imported into Agilent Feature Extraction software for raw data extraction (GEO accession number: GSE165926). Quantile normalization of raw data and subsequent data processing were performed using the R software limma package. Differentially expressed circRNAs between NBT and glioma tissues (fold change ≥ 2 and *P* ≤ 0.05) were considered as statistically significant. Hierarchical clustering was performed to determine the distinguishable expression pattern of circRNAs among samples.

### Cell Culture

The human glioma cell lines U251 (Chinese Academy of Sciences, China), as well as U87, U87MG, LN18, A172, U118, and U138 (American Type Culture Collection, United States) were cultured in DMEM (Dulbecco’s modified Eagle’s medium) (Gibco, Carlsbad, CA, United States) containing 10% fetal bovine serum (Gibco, Carlsbad, CA, United States). Normal human astrocytes HEB were cultured in astrocyte medium. hBMECs (Guangzhou Jennio Biotech, China) were cultured in Endothelial Cell Medium (ScienCell, United States). For the examination of RNA stability, U251 cells were treated with 2 μg/ml actinomycin D (Sigma-Aldrich, China). Inhibitors fasudil hydrochloride and GO6983 were purchased from APExBIO (United States), and NSC305787 was purchased from MCE (China).

The primary glioma cells G10 (derived from glioblastoma, WHO grade IV) and G15 (derived from anaplastic oligodendroglioma, WHO grade III) were dissociated from clinical samples with the Neurosphere Dissociation Kit (P) (Miltenyi Biotec, Germany). Primary glioma cells were cultured in DMEM/F12 supplemented with N2 and B27-A (1:50, Gibco, United States), as well as bFGF and bEGF (20 ng/ml, Gibco, United States). For coculture, hBMEC and G15 or G10 were cultured by a 1:1 mixture of primary glioma cell culture medium and ECM in a six-well transwell system.

For exosome isolation from cell culture supernatant, cells were cultured in serum-free culture medium.

All these cell lines were maintained at 37°C with 5% CO_2_ in a humidified incubator.

### Real-Time Quantitative Reverse Transcription PCR and RNase R Treatment

Total RNA was extracted with TRIzol reagent (Takara, Japan). The quality of isolated RNA was detected using a NanoDrop 2000 spectrophotometer (Thermo Fisher Scientific, United States). RNA was reverse transcribed into cDNA with PrimeScript RT Reagent Kit (Takara, Japan). qRT-PCR was conducted on a Bio-Rad CFX connect system (Bio-Rad, CA, United States) with TB Green Premix Ex Taq (Takara, Japan). The quantification of circRNA and mRNA was normalized to GADPH. For treatment with RNase R (Epicentre, Madison, United States), 1 μg of RNA was digested with 1 unit of RNase R at 37°C for 10 min. The specific primers used are listed in Supplementary Table 2.

### Cell Transfection

The full-length cDNA of circGLIS3 or circGLIS3-FLAG with reversed complementary flanking sequence was amplified and cloned into plasmid pcDNA3.1 (Genepharm, China). Lipofectamine 2000 (Thermo Fisher Scientific, United States) and OptiMEM (Gibco) were used to transfected plasmid into glioma cell lines (for 6 h) or hBMEC (for 4 h). The shRNA targeting the back-splice junction of circGLIS3 (sh-circGLIS3-1, 5′-UCCUGGGAAAGGCUUAUAATT3′; sh-circGLIS3-2, 5′-GGAAAGGCUUAUAACCCACTT-3′) and negative control shRNA (sh-NC) were synthesized and cloned into the LV16 lentiviral vector (Genepharm, China). Cells were incubated with lentivirus and polybrene for 24 h and were selected with puromycin (5 μg/ml, Solarbio, China) for 1 week. The overexpression or knockdown efficiency was confirmed by RT-PCR. The plasmid amplified sequences are shown in Supplementary Figure 2A.

### Migration, Invasion, and Wound Healing Assays

For migration and invasion assays, 3 × 10^4^ treated U87 or U251 cells were suspended in 250 μl serum-free medium and added into the upper chambers (BD Pharmingen, United States) coated with or without Matrigel (Corning, United States), and 750 μl complete medium was added into the bottom chambers. After 24 h, cells on the lower compartment were stained and counted.

For wound healing assay, after 48 h post-transfection of U251 cells, we used a 200-μl pipette tip to scratch lines in the middle of the wells then cultured cells with serum-free medium. The width of wounds was measured and normalized to initial distance.

### Intracranial Xenograft Model

After circGLIS3 overexpression plasmid or shRNA lentiviral transfection, 5 × 10^5^ U87MG cells were injected into the right hemicerebrum of 4-week-old female BALB/c-nu mice. Mice were anaesthetized by pentobarbital sodium during the surgery. Tumor growth was monitored using an *in vivo* imaging system (IVIS Lumina II, Caliper, United States) after the injection of luciferase substrate-D-luciferin (Yeasen, Shanghai, China). Some mice were sacrificed after 16 days, and the brains were removed for further hematoxylin and eosin (HE) and immunohistochemistry (IHC) analysis (*n* = 3 for each group). The remaining mice were monitored for weights and survival time (*n* = 5 for each group). The animal study was reviewed and approved by the Animal Ethics Committee of Southern Medical University and were performed according to the National Institutes of Health (Approval ID: LAEC-2020-090).

### Fluorescence *in situ* Hybridization

Cell climbing pieces were fixed and prehybridized at 55°C for 2 h, then hybridized with specific Cy3-labeled circGLIS3 junction probes (Cy3–5′-TGGTGTGGGTTATAAGC CTTTCCCAGGATTTG-3′-Cy3) (Genepharm, China) at 42°C overnight and dyed with DAPI. The prehybridization buffer and hybridization buffer were obtained using Fluorescent *in situ* Hybridization Kit (Riobo Biotech, Guangzhou, China).

### RNA Sequencing

U251 cells were transfected with sh-circGLIS3 or sh-NC lentivirus for 24 h with polybrene and then treated with puromycin for 7 days. Total RNAs were extracted using TRIzol reagent (Takara, Japan). Two replicates were performed for each group, resulting in a total of six samples. The first step in the workflow involves purifying the poly-A-containing mRNA molecules using poly-T oligo-attached magnetic beads. Following purification, the mRNA is fragmented into small pieces using divalent cations under elevated temperature. The cleaved RNA fragments are copied into first-strand cDNA using reverse transcriptase and random primers. This is followed by second-strand cDNA synthesis using DNA Polymerase I and RNase H. These cDNA fragments then have the addition of a single “A” base and subsequent ligation of the adapter. The products are then purified and enriched with PCR amplification. We then quantified the PCR yield by Qubit and pooled samples together to make a single-strand DNA circle (ssDNA circle), which gave the final library.

DNA nanoballs (DNBs) were generated with the ssDNA circle by rolling circle replication (RCR) to enlarge the fluorescent signals at the sequencing process. The DNBs were loaded into the patterned nanoarrays, and pair-end reads of 100 bp were read through on the BGISEQ-500 platform for the following data analysis study. For this step, the BGISEQ-500 platform combines the DNB-based nanoarrays and stepwise sequencing using Combinational Probe-Anchor Synthesis Sequencing Method.

Raw reads were filtered using SOAPnuke v1.5.2 and were mapped to hg19 using Bowtie2 v2.2.5 (GEO accession number: GSE165859). Differential expression analysis was performed using DESeq. Clustered heat maps were generated in R using the pheatmap package. The differential genes were classified according to the GO and KEGG annotation and classification. Enrichment analysis was generated in R using the phyper package. The *P* value was corrected with FDR. Usually, FDR ≤ 0.01 is regarded as significant enrichment.

### Immunohistochemistry and Immunofluorescence (IF)

Paraffin sections were incubated with primary antibodies at 4°C overnight. For immunohistochemistry (IHC), sections were incubated with secondary antibodies at 37°C for 1 h, stored in HRP-labeled streptavidin solution for 10 min, followed by staining with diaminoaniline (DAB). For immunofluorescence (IF), glioma sections were incubated with Alexa Fluor^®^ conjugated secondary antibodies, then dyed by DAPI. The antibodies and its dilution are shown in Supplementary Table 3.

### RNA Pull-Down

Plasmid and control vector-transfected U251 cell lysates were incubated with biotin-labeled circGLIS3 probe (5′-TGGTGTGGGTTATAAGCCTTTCCCAGGATTTG-3′-Biotin, Genepharm, Shanghai, China). The biotin-labeled RNA complex was pulled down by streptavidin-coated magnetic beads, and the proteins were separated via SDS-PAGE gel. Differentially expressed gel bands were manually cut and digested with sequencing-grade trypsin (Promega, Madison, WI, United States). The digested peptides were analyzed with a Triple TOF 6600 LC-MS system (AB Sciex). The fragment spectra were analyzed using the UniProt database with PEAKS Studio 8.5 (version 8.5, Bioinformatics Solutions Inc., Waterloo, ON, Canada).

### RNA Immunoprecipitation

RNA immunoprecipitation (RIP) was conducted with Magna RIP Kit (Millipore, Billerica, MA, United States) following the manufacturer’s instruments. U251 cell lysate was incubated with magnetic beads which were conjugated with anti-p-Ezrin(T567) (ab76247, Abcam), anti-Ezrin (ab40839, Abcam), or positive control anti-SNRNP70 and negative control anti-IgG antibody (Millipore, MA, United States) at 4°C for 6 h. The beads were washed. Then, immunoprecipitated RNA and protein were purified and enriched to detect the target RNAs and proteins by qRT-PCR and Western blot.

### Western Blot Analysis

The total protein was exacted with RIPA buffer containing protease inhibitors and phosphatase inhibitors (CWBio, Beijing, China). Products were separated by SDS-PAGE, then electransferred onto a PVDF membrane (Bio-Rad, CA, United States). The membranes were blocked with 5% BSA and incubated with primary antibodies at 4°C overnight and then incubated with secondary antibodies at room temperature for 1 h. The antibodies and its dilution are shown in Supplementary Table 3. Finally, the bands were visualized using Image Lab software with HRP Substrate (Millipore, United States).

### Statistical Analysis

Statistical analyses were performed by SPSS 20.0 (IBM, United States) and GraphPad Prism 7.0 (GraphPad Software Inc., United States). Data were shown as mean ± standard error of mean (SEM). The differences between two groups were assessed by unpaired two-tailed Student’s *t*-test. Comparisons between multiple groups were performed by one-way or two-way analysis of variance ANOVA with Bonferroni *post hoc* tests. CircGLIS3 expression heterogeneity in glioma tissues was assessed with the χ^2^ test. The survival rates were evaluated by Kaplan–Meier method and tested by log-rank test. Pearson correlation analysis was performed by simple linear regression. *P*-value < 0.05 was considered as statistically significant.

## Results

### CircGLIS3 Is Highly Expressed in High-Grade Glioma

**FIGURE 1:**
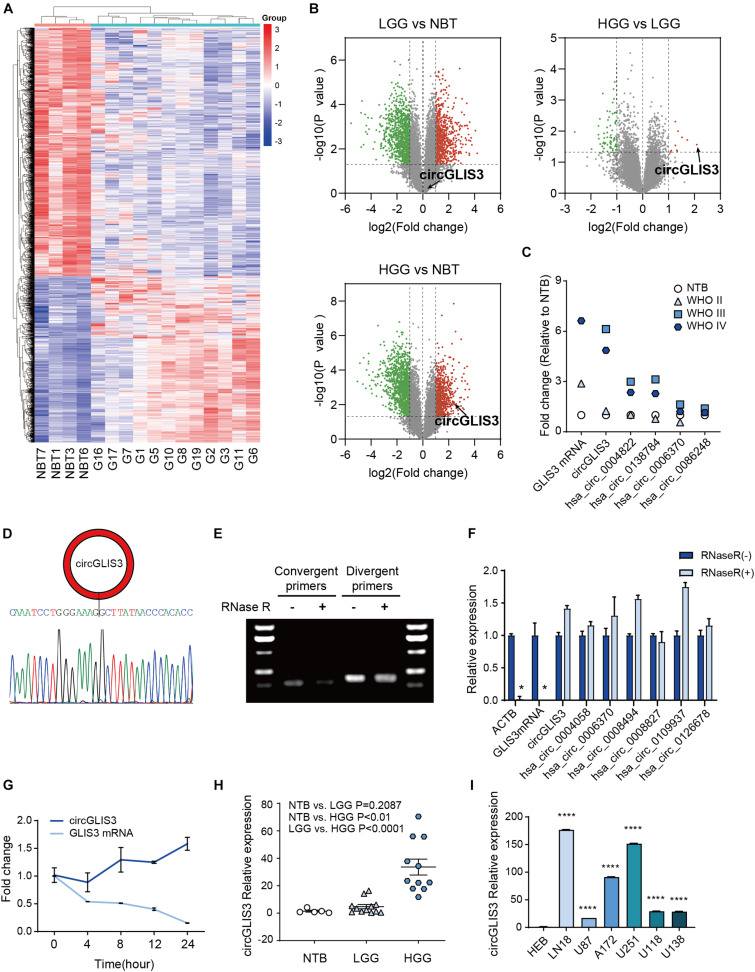
CircGLIS3 is highly expressed in HGG. **(A)** The cluster heat map from microarray data (*P* < 0.05) shows circRNAs with different expression patterns among 16 tissue samples. **(B)** The volcano plot for the differently expressed circRNAs among NBT (*n* = 4), LGG (*n* = 4), and HGG (*n* = 8), circGLIS3 is signed out. **(C)** Quantification fold change of five circRNAs originated from *GLIS3* in microarray data. The GLIS3 mRNA quantification is from Song et al. (2016), NBT (*n* = 4), WHO grade II (*n* = 4), WHO grade III (*n* = 3), and WHO grade IV (*n* = 5). **(D)** Sanger sequencing of circGLIS3 qPCR divergent primer amplification products. Sequences can blast to the circGLIS3 junction. **(E)** Northern blot of circGLIS3 qPCR amplification products with or without RNase R treatment. **(F)** RT-PCR of RNAs in U87 cells with or without RNase R treatment. **(G)** qPCR for the abundance of circGLIS3 and linear GLIS3 mRNA in U87 cells treated with actinomycin D at indicated time points. **(H)** RT-PCR detection of circGLIS3 in NBT (*n* = 5), LGG (*n* = 12), and HGG (*n* = 11). **(I)** RT-PCR detection of circGLIS3 in HEB and glioma cell lines. The experiments were repeated for three times with three repetitions for each group. The data are presented as the mean ± SEM, *P < 0.05, **P < 0.01, ***P < 0.001, ****P < 0.0001.

We performed circRNA microarray analysis from four NBTs, four WHO grade II gliomas (LGG), three WHO grade III gliomas, and five WHO grade IV glioblastomas (HGG). As shown in Figure 1A, 13,257 circRNAs were detected using probes designed for 13,617 human circRNAs from circBase^1^ (Glažar et al., 2014) and other literatures, revealing differential circRNA expression patterns in gliomas and NBT.

Among the candidates, we found hsa_circ_0002874, which was circularized by exon 2 of the *GLIS3* gene (we termed hsa_circ_0002874 as circGLIS3 below) and was highly expressed in HGG samples compared with LGG and NBT (*P* = 0.0271 and 0.0090; Figure 1B and Supplementary Table 4), in accordance with Song et al. (2016). CircGLIS3 expression was both elevated in WHO grade III and WHO grade IV of HGG (*P* = 0.0039 and 0.0430) (Figure 1C). Furthermore, among the five circRNAs originated from *GLIS3* (circGLIS3, hsa_circ_0004822, hsa_circ_0138784, hsa_circ_0006370, and hsa_circ_0086248) measured by microarray analysis, the circGLIS3 expression level in glioma was increased most (Figure 1C), similarly with the GLIS3 mRNA expression level according to Song et al. (2016), indicating that circGLIS3 might be specifically regulated in HGG.

Next, we examined circGLIS3 characteristics. Sanger sequencing with the circGLIS3 divergent primers was performed to verify the back-spliced structure of circGLIS3 (Figure 1D). Treatment with RNase R followed by Northern blot or RT-PCR in U87 cells showed that circGLIS3 is resistance to RNase R digestion (Figures 1E,F). Moreover, treatment with actinomycin D demonstrated that circGLIS3 is more stable than *GLIS3* linear mRNA (Figure 1G). We then measured the circGLIS3 expression level by RT-PCR and also found a universal upregulation of circGLIS3 in HGG tissues and glioma cell lines (Figures 1H,I).

### CircGLIS3 Enhances Migration and Invasion of Glioma Cells *in vitro* and *in vivo*

**FIGURE 2:**
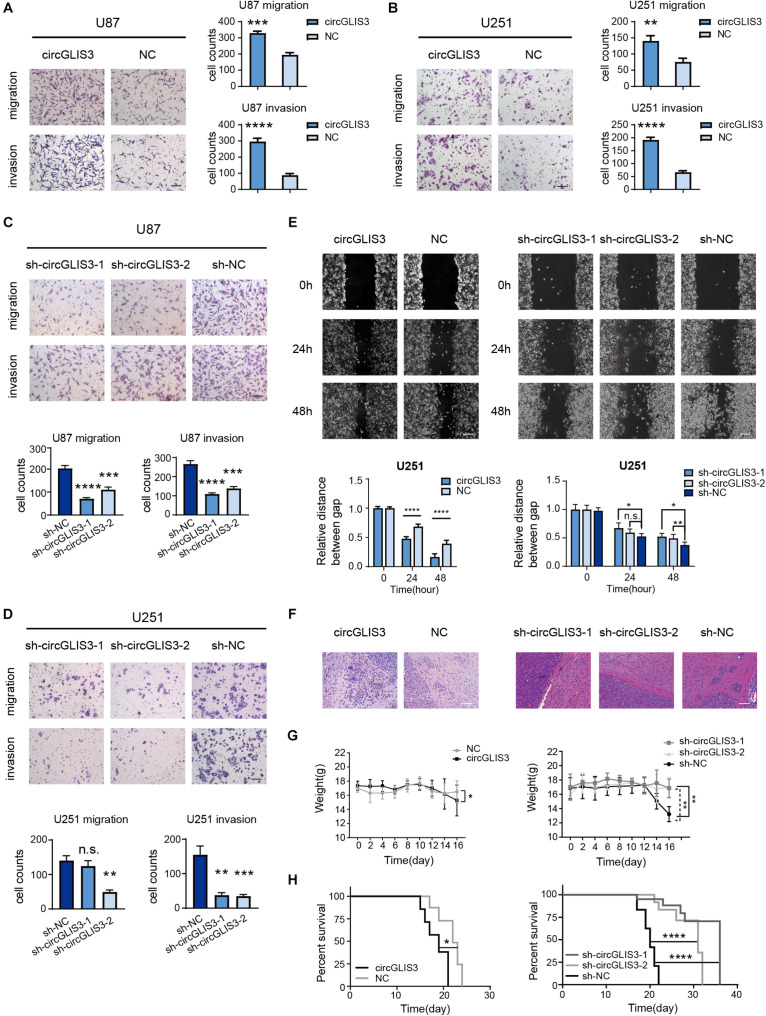
CircGLIS3 enhances migration and invasion of glioma cells *in vitro* and *in vivo*. **(A)** Transwell migration and invasion assay of U87 and **(B)** U251 cells transfected with circGLIS3 plasmid, 3 × 10^4^ cells were seeded in the upper chamber (magnification, ×100; scale bar, 100 μm). **(C)** Transwell migration and invasion assay of U87 and **(D)** U251 cells transfected with sh-circGLIS3 lentivirus, 3 × 10^4^ cells were seeded in the upper chamber (magnification, ×100; scale bar, 100 μm). **(E)** Wound healing assays of treated U251 cells (magnification, ×100; scale bar, 100 μm). The experiments were repeated for three times with three repetitions for each group. **(F)** Hematoxylin eosin staining of 16 days intracranial xenograft model (*n* = 3 for each group) (scale bar, 100 μm). **(G)** Weights and **(H)** Kaplan–Meier survival curve of the intracranial xenograft model (*n* = 5 for each group). The data are presented as the mean ± SEM, *P < 0.05, **P < 0.01, ***P < 0.001, ****P < 0.0001.

Next, we aimed to explore the biological function of circGLIS3 in HGG. The pcDNA3.1 vector comprising the full length of circGLIS3 with a flanking sequence at the 5′ and 3′ ends was designed to specifically upregulated circGLIS3 expression without influencing GLIS3 mRNA expression (Supplementary Figures 1A,B). Cell function assays were performed after transfecting vectors into glioma cell lines U87 and U251. As shown in Figures 2A,B, elevating circGLIS3 in U87 and U251 cells increased the number of migrating or invading cells through transwell chambers and the Matrigel-coated transwell chambers. Opposite results were observed in U87 and U251 after knocking down circGLIS3 by lentivirus carrying circGLIS3 short hairpin RNAs (shRNA), which proved the active effect of circGLIS3 on migration and invasion in glioma (Figures 2C,D and Supplementary Figure 1C). Wound scratching assay also showed the improving moving abilities in U251 cells by circGLIS3 (Figure 2E). While changing the circGLIS3 expression level in glioma cells had no effect on glioma proliferation, apoptosis, and vascular mimicry (data not shown).

To investigate the influence of the circGLIS3 expression level on tumor progression *in vivo*, circGLIS3 overexpression plasmid or sh-circGLIS3 lentivirus-transfected U87MG cells were used to form intracranial xenografting models. Tumors formed by upregulated or downregulated circGLIS3 U87MG and control U87MG cells had similar sizes (Supplementary Figures 2A–D), consistent with *in vitro* experiment results that circGLIS3 has no influence on glioma proliferation. The HE staining of intracranial tumors showed that, compared to the sh-NC group glioma surrounded by several satellites, there are few satellites around a smooth tumor borders in the sh-circGLIS3 groups (Figure 2F). Each satellite tumor showed a central vessel and peripheral glioma cells. While in the circGLIS3 group, single or several glioma cells invade into brain parenchyma, causing a rougher border of glioma compared with the NC group (Figure 2F). Likewise, circGLIS3 was associated with a decreased weight loss in nude mice (*P* = 0.0284 of the circGLIS3 group, *P* = 0.0068 and 0.0010 of the sh-circGLIS3 group) (Figure 2G). The medium survival time of the circGLIS3 group (19 days) was shorter than the NC group (22 days) (*P* < 0.05), and the medium survival time of the sh-circGLIS3 groups (36 and 31 days) was longer than the time of the sh-NC mice group (20 days) (both *P* < 0.0001) (Figure 2H). These results showed that a higher circGLIS3 expression level was correlated with malignant phenotype in glioma.

### CircGLIS3 Directly Binds With p-Ezrin(T567) and Elevates p-Ezrin(T567) Level in Glioma

**FIGURE 3:**
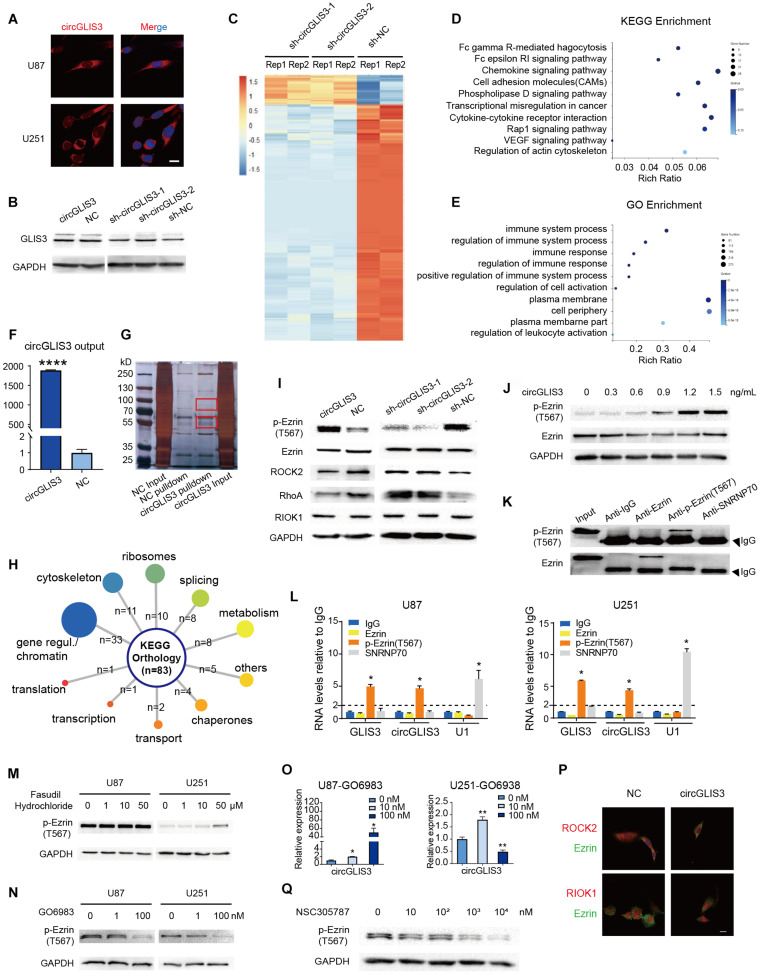
CircGLIS3 directly binds with p-Ezrin(T567) and elevates the p-Ezrin(T567) level in glioma. **(A)** Fluorescence *in situ* hybridization (FISH) assay with Cy3-labeled circGLIS3 probe (magnification, ×640; scale bar, 10 μm). **(B)** Western blot of GLIS3 protein after the upregulated or downregulated circGLIS3 level in U251 cells. **(C)** RNA-Seq analysis of U251 cells transfected by sh-circGLIS3 or sh-NC lentivirus. **(D)** KEGG and **(E)** GO enrichment analysis in U251 cells. **(F)** RNA pull-down experiments with U251 extract. Pull-down probe efficiency was determined by RT-PCR. **(G)** RNA pull-down experiments with U251 extract. Specific bands were identified by mass spectrometer. **(H)** Summary of circGLIS3 binding proteins according to KEGG. **(I)** Western blot detection after upregulated or downregulated circGLIS3 in U251. **(J)** Western blot detection of p-Ezrin(T567) in U87 transfected by concentration gradient circGLIS3 plasmid. **(K)** Western blot of RIP lysate by anti-Ezrin, anti-p-Ezrin(T567), anti-IgG, and anti-SNRNP70 antibodies. **(L)** RT-PCR analysis of Ezrin, p-Ezrin(T567), IgG-negative control, and SNRNP70-positive control immunoprecipitated RNA of U87 (left) and U251 (right). **(M)** Western blot of p-Ezrin(T567) in U87 and U251 after being treated with DMSO or fasudil hydrochloride for 2 days. **(N)** Western blot of U87 and U251 after treated with GO6983 for 2 days. **(O)** RT-PCR of U87 and U251 after being treated with GO6983 for 2 days. **(P)** Confocal microscope image of Ezrin and ROCK2 or RIOK1. Scale bar, 5 μm. **(Q)** Western blot of p-Ezrin(T567) in U87 after being treated with NSC305787 for 9 h. The results are presented as the mean ± SEM of biological triplicate assays. *P < 0.05, **P < 0.01, ****P < 0.0001.

We sought to determine the possible molecular mechanism of circGLIS3 in HGG. As previous studies reported that circRNA function relates with its cellular location, we designed circGLIS3 junction probe with Cy3 modification and performed RNA fluorescence *in situ* hybridization (FISH) assay in two glioma cell lines U87 and U251. As shown in Figure 3A, abundant cytoplasmic circGLIS3 were observed.

Firstly, because circGLIS3 is cytoplasmic and changing the circGLIS3 expression level had no effect on the expression of GLIS3 mRNA and protein (Supplementary Figures 1B,C and Figure 3B), we excluded the possibility that circGLIS3 acts by influencing its parental gene transcription or translation. Then, we analyzed the translational potential of circGLIS3 on circRNADb^2^ (Chen et al., 2016) and found a putative open reading frame (ORF) and two internal ribosomal entrance sites (IRES) within the circGLIS3 sequence. So we designed a circGLIS3-FLAG plasmid with addition of a separating FLAG tag sequence to both sides of the circGLIS3 junction, referring to Zhang et al. (Yang et al., 2018) (Supplementary Figures 3A,B). However, a predicted 139-aa protein band was not observed by Western blot in transfected U87 and U251 cell lines (Supplementary Figures 3C,D).

We further performed RNA sequencing of sh-circGLIS3 or sh-NC lentivirus-transfected U251 cells. Results showed a transcriptional inhibitory effect on cell adhesion and cell membrane after downregulating the circGLIS3 expression level (Figures 3C–E). Next, we performed RNA pull-down assays in plasmid-transfected U251 cells with biotin-labeled circGLIS3 junction probe. Specific bands around 55 and 100 kDa were identified by mass spectrometry (Figures 3F,G). Compared with the negative control group, 83 proteins with enrichment were detected from circGLIS3-overexpressing cell lysates, most of which were involved in regulation of genes/chromosomes (39.8%) and assembly of cytoskeleton (13.3%) (Figure 3H). Considering its cytoplasmic location and the biological function on movement, we focused on cytoskeleton proteins, especially the most enriched protein Ezrin.

Because phosphorylation of Thr567 (T567) is crucial for Ezrin conformation and activation, we also wondered whether circGLIS3 affects Ezrin phosphorylation status. We transfected U251 cells with circGLIS3 plasmid or shRNA lentivirus and observed a correlated phosphorylation level alteration on Thr567 in Ezrin but no effect on total Ezrin expression (Figure 3I). In addition, circGLIS3 raised the Ezrin T567 phosphorylation level in a dose-dependent manner in U87 (Figure 3J). Given the fact that circGLIS3 elevated p-Ezrin(T567), we performed RIP assays to confirm the interaction between Ezrin and circGLIS3. Surprisingly, phosphorylated Ezrin(T567), but not Ezrin protein, could specifically bind to circGLIS3 (Figures 3K,L).

Since ROCK2 and PKC, two reported Ezrin kinases on threonine 567, were not detected by circGLIS3 pull-down and the following mass spectrometry, and the only threonine kinase RIOK1 detected by mass spectrometry is in the nucleus rather than cytoplasm or membrane, we supposed that circGLIS3 directly binds to p-Ezrin(T567) and maintains its active condition rather than form a complex with Ezrin and kinases to enhance Ezrin phosphorylation. Firstly, we treated U87 and U251 cells with threonine kinase inhibitors: (i) fasudil hydrochloride, a non-specific ROCK inhibitor (IC50 of 0.158 μM for ROCK2) which also has an inhibitory effect on PKC (IC50 of 12.30 μM for PKC), or (ii) GO6983, a pan-PKC inhibitor. Treatment with fasudil hydrochloride did not reduce the 567 position threonine residues of Ezrin phosphorylation in U87 cells and even elevated p-Ezrin(T567) in U251 cells (Figure 3M). GO6983 could reduce the p-Ezrin(T567) level in U87 and U251 cells, but disappointedly upregulated the circGLIS3 level (Figures 3N,O). It seemed that Ezrin was mainly phosphorylated by PKC in glioma. Next, we used confocal microscopy to observe the cellular co-localization of Ezrin with ROCK2 or RIOK1. Vector-mediated circGLIS3 overexpression in U87 cells showed no changed co-localized degree (Figure 3P) and no effect on RIOK1 nucleus localization. Then, Western blot was carried out to measure whether changing the circGLIS3 expression level would reversely influence the expression of threonine kinase. Results showed that circGLIS3 can inhibit the RhoA/ROCK2 pathway and have no effect on the RIOK1 protein level (Figure 3I).

### Elevated p-Ezrin(T567) Is Related to High Grade and Poor Prognosis of Glioma Patients

**FIGURE 4:**
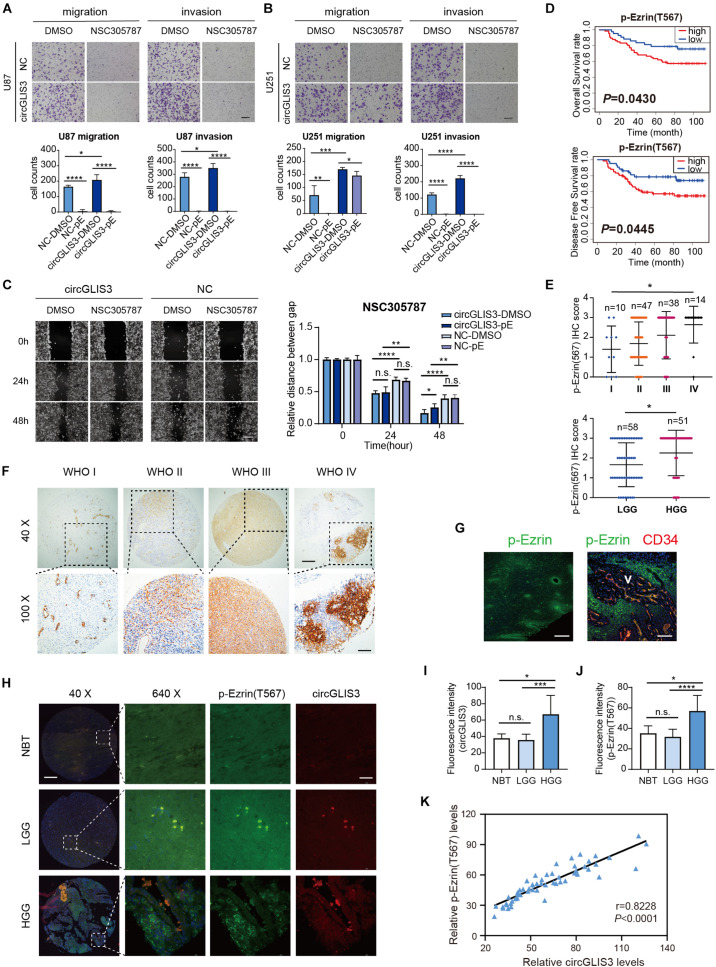
Elevated p-Ezrin(T567) is related to high grade and poor prognosis of glioma patients. **(A)** Transwell migration and invasion of U87 and **(B)** U251 cells, 3 × 10^4^ cells were seeded and treated with 1 μM NSC305787 (noted as pE group) or DMSO for 24 h (magnification, ×100, scale bar, 100 μm). **(C)** Wound healing assay of U251 cells after being transfected with plasmid for 2 days and treated with 0.5 μM NSC305787 (noted as pE group) or DMSO (magnification, ×100; scale bar, 100 μm). **(D)** Kaplan–Meier survival curve analysis and **(E)** dot distribution graph of p-Ezrin(T567) IHC staining score were shown within 109 glioma patients of different WHO grades. **(F)** IHC staining of p-Ezrin(T567) in 109 different grades of gliomas. Vascular endothelial cells also showed a strong positive stain with p-Ezrin(T567) (magnification, ×40 (upper), scale bar, 200 μm; ×100 (lower), scale bar, 100 μm). **(G)** Immunofluorescence stain of p-Ezrin(T567) and CD34 in glioma tissue (scale bar, left: 300 μm, right: 80 μm). **(H)** Fluorescence *in situ* hybridization of circGLIS3 and immunofluorescence stain of p-Ezrin(T567) in 58 NBT and glioma samples (NBT = 3, LGG = 9, HGG = 46) [magnification, ×40 (left), scale bar, 200 μm; ×640 (right), scale bar, 40 μm]. **(I)** Fluorescence intensity analysis of circGLIS3 *in situ* stain in 58 samples. **(J)** Fluorescence intensity analysis of p-Ezrin(T567) *in situ* stain in 58 samples. **(K)** A positive correlation was shown between circGLIS3 and p-Ezrin(T567) expression in 58 samples using Pearson correlation analysis. The results are presented as the mean ± SEM. *P < 0.05, **P < 0.01, ***P < 0.001, ****P < 0.0001.

We further explored the function of p-Ezrin(T567) in gliomas. Ezrin inhibitor NSC305787 mimics the Ezrin morpholino phenotype and binds directly to Ezrin, which can inhibit the Ezrin T567 phosphorylation (Figure 3Q). Transwell assay showed that glioma cell motion could be restrained when inhibiting phosphorylation of the T567 residue of Ezrin with NSC305787, which is also observed in glioma cells after transfecting sh-circGLIS3 lentivirus (Figure 4A). Next, we used NSC305787 to rescue the effects of circGLIS3 on glioma cells. Migration and invasion assays showed that NSC305787 can partially cancel out the influence of circGLIS3 (Figures 4A,B; 1 μM NSC305787 for 24 h), while this efficiency was not obvious in wound healing assays (Figure 4C; 0.5 μM NSC305787 for 48 h; 1 μM NSC305787 treated for 48 h could cause U251 cell rounding and death).

To figure out the correlation between p-Ezrin(T567) expression and clinical-pathological features in glioma patients, we conducted tissue microarray analysis in different grades of 109 glioma tissues. The Kaplan–Meier survival curve showed that a high level of p-Ezrin(T567) is related with poor prognosis (OS *P* = 0.0430, DFS *P* = 0.0445) (Figure 4D). Besides, we found that the expression of p-Ezrin(T567) was also positively correlated with glioma grade (*P* = 0.0116 and 0.0162) (Figure 4E,F), but not with sexual (*P* = 0.4086), age (*P* = 0.0585), and glioma recurrence (*P* = 0.1263) (Table 1). Interestingly, we also find a perivascular distribution pattern of p-Ezrin(T567) in glioblastoma (Figures 4F,G), similarly with 87MG perivascular distribution of glioma satellite in nude mice (Figure 2F).

**TABLE 1:** Correlation between p-Ezrin(T567) expression and clinical–pathological features in 109 glioma patients.

		Low (score = 0, 1)	High (score = 2, 3)	Total	Chi-square	*P*
Total		43	66	109		
Sexual	Male	26	45	71	0.6827	0.4086
	Female	17	21	38		
Age	<45	30	34	64	3.578	0.0585
	≤45	13	32	45		
WHO grade	I	5	5	10	6.366 (for trend)	0.0116*
	II	24	24	48		
	III	12	25	37		
	IV	2	12	14		
Grade	Low	29	29	58	5.777	0.0162*
	High	14	37	51		
Recurrence	Yes	21	42	63	2.338	0.1263
	No	22	24	46		

*P < 0.05.

To figure out the correlation of circGLIS3 expression with p-Ezrin(T567) levels in glioma clinical samples, we stained both circGLIS3 and p-Ezrin(T567) on another tissue microarray with 58 NBT and glioma tissues. As shown in Figure 4H, only in HGG could a specific positive co-stain of circGLIS3 with p-Ezrin(T567) be observed. Fluorescence intensity analysis of *in situ* stain also showed that circGLIS3 and p-Ezrin(T567) are up-expressed in HGG (Figures 4I,J). Besides, Pearson correlation analysis revealed that the expression of circGLIS3 was correlated with the level of p-Ezrin(T567) in glioma tissues (Figure 4K).

### CircGLIS3 Is Secreted Into Glioma Microenvironment and Participates in Tumor Angiogenesis

**FIGURE 5:**
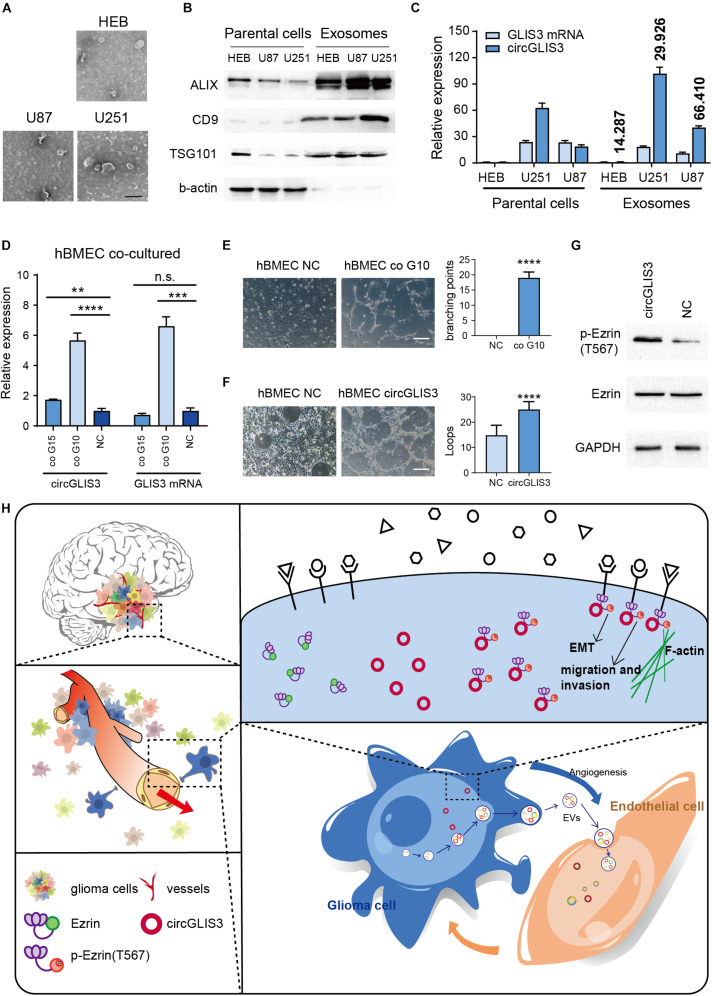
CircGLIS3 is secreted into the microenvironment and participates in tumor angiogenesis. **(A)** Electron microscopy images of exosomes isolated from HEB, U87, and U251 cell culture supernatant (scale bar, 200 nm). **(B)** Western blot of exosomal markers and b-actin control in HEB, U87, and U251 cells and exosomes. **(C)** RT-PCR for the level of circGLIS3 and its linear counterparts in HEB, U87, and U251 cells (left) and corresponding exosomes (right); data were normalized to GAPDH. Fold enrichment of circGLIS3 compared to GLIS3 mRNA in each cell line is calculated according to 2^–ΔΔCT^ and shown above the column. **(D)** RT-PCR of hBMEC after being cocultured with G15 and G10 for 5 days. Cells were cultured by a 1:1 mixture of primary glioma cell culture medium and ECM medium. **(E)** Tube formation assay of 3 × 10^5^ hBMEC in a 24-well plate after being cocultured with G10 for 7 days (upper) or **(F)** after being transfected with circGLIS3 plasmid (300 ng/ml) for 48 h (lower) (scale bar, 150 μm). **(G)** Western blot of p-Ezrin(567) and Ezrin in hBMEC after transfected with the circGLIS3 plasmid for 48 h. **(H)** Diagram of circGLIS3 in glioma progression. The results are presented as the mean ± SEM of biological triplicate assays. **P < 0.01, ***P < 0.001, ****P < 0.0001.

Lasda and Parker (2016) reported that circRNA can be selectively secreted out by exosomes, so we wondered whether circGLIS3 can be excreted through exosomes and play a role in glioma microenvironment. Human astrocyte cell line HEB and glioma cell lines U87 and U251 were cultured in serum-free culture medium. The culture supernatants of HEB, U87, and U251 were collected for exosome isolation (Figures 5A,B). RT-PCR was conducted to measure the expression levels of circGLIS3 and GLIS3 mRNA in exosomes (Figure 5C). Results showed that circGLIS3 was more abundant in glioma cell exosomes compared with HEB exosomes. Besides, the ratio of circGLIS3 to GLIS3 mRNA within exosomes is increased compared with the cellular RNA ratio, indicating a selective secretion of circGLIS3 in glioma exosomes (Figure 5C).

Due to the invasiveness and perivascular characteristics of glioma cells in nude mice model HE stain (Figure 2F) and the perivascular stain distribution of p-Ezrin(T567) in glioma tissue IHC and IF stain (Figures 4F–H), we discussed whether and how the exosome-derived circGLIS3 of glioma would influence vascular endothelial cells. We cocultured hBMECs with two primary glioma cells G10 and G15, derived from glioblastoma (WHO grade IV) and anaplastic oligodendroglioma (WHO grade III), separately. Both G10 and G15 revealed a trend of increasing circGLIS3 level in hBMEC after being cocultured for 5 days without direct contact (Figure 5D).

To figure out the function of circGLIS3 in hBMEC, we conducted tube formation assay with hBMEC which was cocultured with G10 for 5 days or transfected with circGLIS3 plasmid and found that both treatment can increase vascularization in hBMEC (Figures 5E,F). Western blot following transfection with plasmid showed that circGLIS3 could also promote the phosphorylation level of Ezrin T567 in hBMEC (Figure 5G). Taken together, our data suggest that circGLIS3 might be packaged into exosomes and be absorbed by brain vascular endothelial cells for the purpose of tumor angiogenesis in glioma (Figure 5H).

## Discussion

Gene regulation might be far more important than gene function since exons only cover 2.94% of the human genome (Encode Project Consortium., 2012). Non-coding RNAs can function as regulators widely participating in physiopathology processes. Spatial and time-specific expression of non-coding RNA brings complexity and precision to regulation. As a rising star of non-coding RNA, circRNAs have attracted wide attention since circRNA CDR1as was found to be functional by sponging miR-7 7 years ago (Hansen et al., 2013). Structurally, a circRNA sequence without 5′ Gcp cap and 3′ polyadenylate tail makes it more stable and could be detected as a biomarker (Rajappa et al., 2020). Moreover, the circRNA loop structure tends to form internal secondary and tertiary structures, which brings complicacy to predict and discover interactions between circRNAs and other biomolecules. Experimental measurements are necessary to study the circRNA–protein interaction, owing to the limited efficiency of bioinformatics analyses (Huang et al., 2020). Above all, the expression and regulatory function of most circRNAs still need clarification.

CircGLIS3 is originated from gene *GLIS3*, which encodes a nuclear protein with dual transcriptional influence. A few studies have referred to the correlation of the *GLIS3* transcriptional level with higher grade of gliomas and with poor outcome of ependymoma (Lukashova-v et al., 2007; Cooper et al., 2010), but the function and mechanism have not been discovered. An abnormal expression of circGLIS3 in HGG was only mentioned by Song et al. (2016). He performed RNA-seq in 46 gliomas and normal brain samples to test a new computational pipeline for detecting circRNAs without investigating circRNA functions. Our circRNA microarray analysis also found circGLIS3 merely upregulated in high-grade glioma. Here, we demonstrate that circGLIS3 plays an important role in glioma progress. Functionally, the circGLIS3 expression level was positively related to glioma invasion. Overexpression of circGLIS3 could increase glioma migration and invasion abilities *in vitro*, while downregulated circGLIS3 in glioma cells displayed an opposite effect. Meanwhile, intracranial glioma nude mouse models showed that the invasion phenotype of glioma cells is positively correlated with the circGLIS3 expression level. Mechanistically, we demonstrate the direct interaction with circGLIS3 and p-Ezrin(T567). CircGLIS3 could increase the phosphorylation level of Ezrin T567 in glioma cells and then increase glioma cell motility. Finally, we preliminarily discussed whether circGLIS3 participated in regulating the glioma microenvironment. We found that circGLIS3 can be excreted by glioma through exosomes, elevate the circGLIS3 level of hBMEC, and lead to an angiogenesis effect in hBMEC. Collectively, circGLIS3 contributes to high-grade glioma invasiveness via directly binding with p-Ezrin(T567) and contributes to glioma angiogenesis via exosome secretion.

Notably, unlike the widespread studies on the circRNA sponging mechanism, our results showed that circGLIS3 functions in regulating posttranslational modification in protein Ezrin. As one of the non-coding RNAs, circRNA somehow shares similar functions within lncRNA. Since Wang et al. found out that lnc-DC can prevent dephosphorylation of STAT3 Y705 by SHP1 in human dendritic cells in 2014, scattered studies clarified the role of non-coding RNAs on protein posttranslational modification (Wang et al., 2014, 2018; Liu et al., 2015). However, due to the complex structure of circRNA, there are relatively few studies on the interaction between circRNA and proteins overall. Only recently was a circRNA named CDR1as found out, preventing p53 from ubiquitination in gliomas (Lou et al., 2020). Unfortunately, considering that the correct and complete domains are vital in transforming active conditions of Ezrin, and accurately nucleotide sequence is necessary in confirming the circRNA stem-loop structure, we did not design truncation Ezrin protein and mutation circGLIS3 in our study to find out a precise binding domain in which circGLIS3 blocked Ezrin from dephosphorylation.

Besides, our results also showed the relation between Ezrin T567 phosphorylation and glioma. Ezrin has been widely studied in tumor metastasis (Zhan et al., 2019; Fan et al., 2021). Ezrin mRNA expression level, total protein level, and p-Ezrin(F353) level are reported to contribute to glioma malignant phenotype (Wick et al., 2001; Mao et al., 2014; Liu et al., 2020). However, the 567 position threonine residue phosphorylation status of Ezrin in glioma has not been studied yet. We found that a higher IHC score of p-Ezrin(T567) might correspond to higher glioma WHO grade and to a shorter lifetime for patients. Besides, Ezrin is a cytoskeletal protein rather than a canonical RBP. Previous studies showed how non-coding RNA impacted Ezrin on a transcriptional level. It is reported that microRNA-204 and circARHGAP12 regulate Ezrin through binding to the 3′-UTR of Ezrin mRNA (Mao et al., 2014; Fan et al., 2021). In this study, we confirmed that circGLIS3 can regulate Ezrin biological function in glioma through directly binding with p-Ezrin(T567).

Another key finding obtained from the current study was that circGLIS3 regulates the glioma microenvironment. Growing evidence showed that multiple tumors changed their microenvironment through exosome secretion. Messages containing mRNA, microRNA, proteins, and circRNA are selectively packaged and conveyed through the exosome (Lasda and Parker, 2016). Lucero et al. (2020) revealed that glioma-derived miRNA-containing extracellular vesicles could induce glioma angiogenesis, and Ding et al. (2020) found out that exosome-derived circNFIX could enhance temozolomide resistance in glioma. Our research detected that glioma cells can selectively secrete circGLIS3 via exosome. Besides, since we observed the perivascular characteristic of glioma cells in nude mouse model HE stain and tissue microarray IHC and IF stain, we discussed the influence of glioma on endothelial cells. Glioma cells cocultured with hBMEC can actually upregulate circGLIS3 in endothelial cells and promote hBMEC angiogenesis. Moreover, circGLIS3 functioned in hBMEC also by modulating Ezrin phosphorylation.

While further researches are required, the potential value of circGLIS3 as a glioma biomarker and a therapeutic target cannot be ignored. These findings suggest that despite many obstacles, there are thousands of functional circRNAs remaining to be found in regulating proteomics, as well as quantities of novel and efficient experimental and bioinformatic approaches remaining to be produced in studying circRNA–protein interactions. These findings also suggest the potential of circRNAs in regulation of the tumor microenvironment and as targets for diagnosis and therapy.

## Conclusion

In summary, our study demonstrates that circGLIS3 is upregulated in high-grade glioma and contributes to the invasion and angiogenesis of glioma via modulating Ezrin T567 phosphorylation.

## Data Availability Statement

The datasets presented in this study can be found in online repositories. The names of the repository/repositories and accession number(s) can be found in the article/Supplementary Material.

## Ethics Statement

The studies involving human participants were reviewed and approved by the Clinical Research Ethics Committee of Zhujiang Hospital, Southern Medical University. Written informed consent to participate in this study was provided by the participants or their legal guardian/next of kin. The animal study was reviewed and approved by the Animal Ethics Committee of Southern Medical University.

## Author Contributions

YK and JWa contributed to the design of the study. YL and JC contributed to perform all experiments and wrote the manuscript. ZC contributed to analyze the data of the microarray. XX contributed to collection of the glioma tissues and NBT. JWe contributed to analyze the data of RNA-seq. YZ, YM, and YL contributed to analyze the experiments data. All authors contributed to the article and approved the submitted version.

## Conflict of Interest

The authors declare that the research was conducted in the absence of any commercial or financial relationships that could be construed as a potential conflict of interest.

## Publisher’s Note

All claims expressed in this article are solely those of the authors and do not necessarily represent those of their affiliated organizations, or those of the publisher, the editors and the reviewers. Any product that may be evaluated in this article, or claim that may be made by its manufacturer, is not guaranteed or endorsed by the publisher.

## Footnotes

1
http://www.circbase.org/

2
http://reprod.njmu.edu.cn/cgi-bin/circrnadb/circRNADb.php
