# Supplementary material for: CircGLIS3 Promotes High-Grade Glioma Invasion via Modulating Ezrin Phosphorylation
Source: Front Cell Dev Biol. 2021 Sep 3;9:663207. doi: 10.3389/fcell.2021.663207 (PMC8446459; doi:10.3389/fcell.2021.663207)
Supplement: Supplementary file 1 [file Table_1.docx]

Supplementary Material

## Supplementary Figures


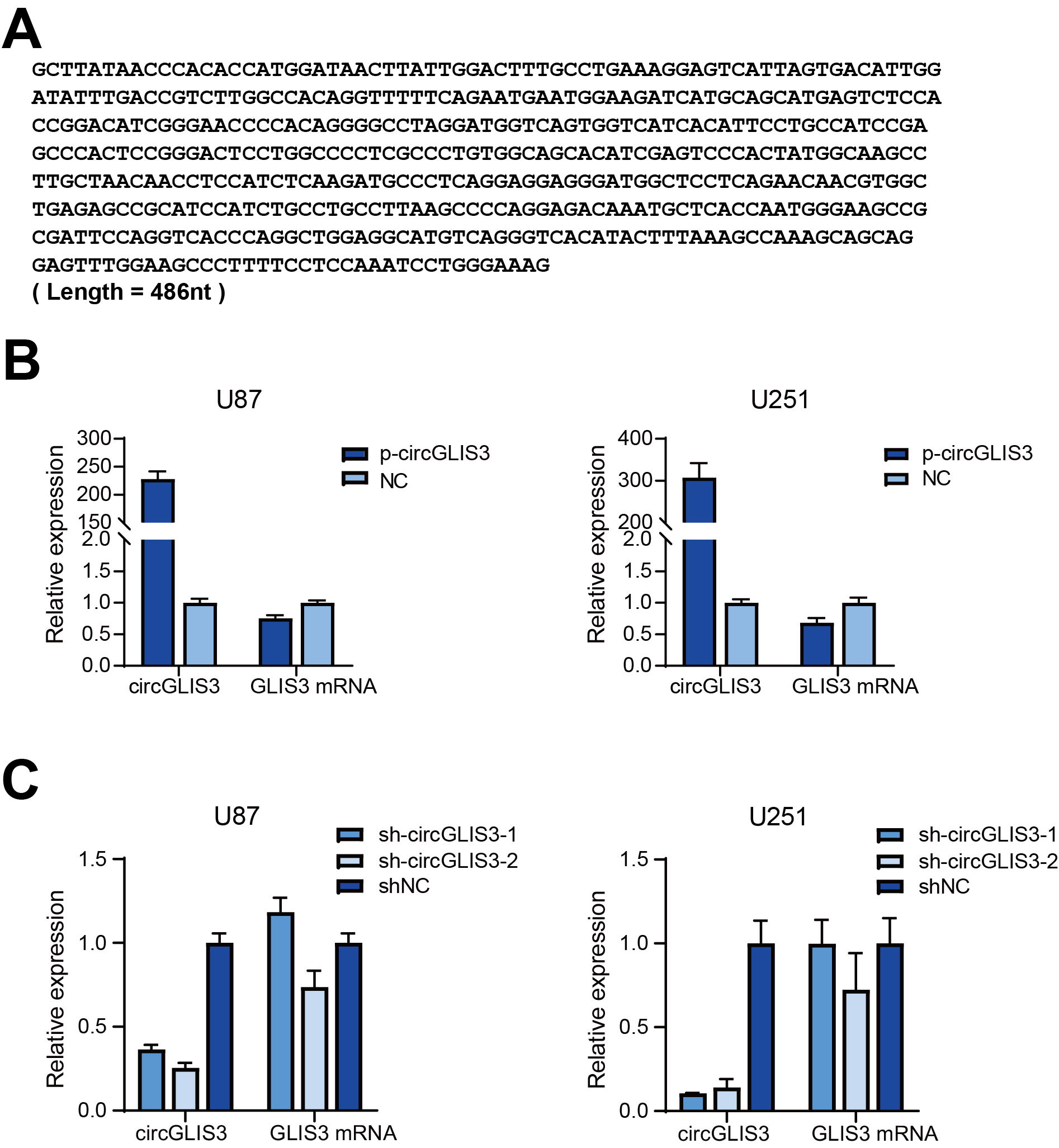


**Supplementary Figure 1.** **Up-regulate or knockdown circGLIS3 in glioma cells.**(**A**) Total sequence of circGLIS3. (**B**) RT-PCR of circGLIS3 expression level in circGLIS3 plasmid transfected U87 and U251 (mean ± SEM). (**C**) RT-PCR of circGLIS3 expression level in sh-circGLIS3 lentivirus transfected U87 and U251.(mean ± SEM).


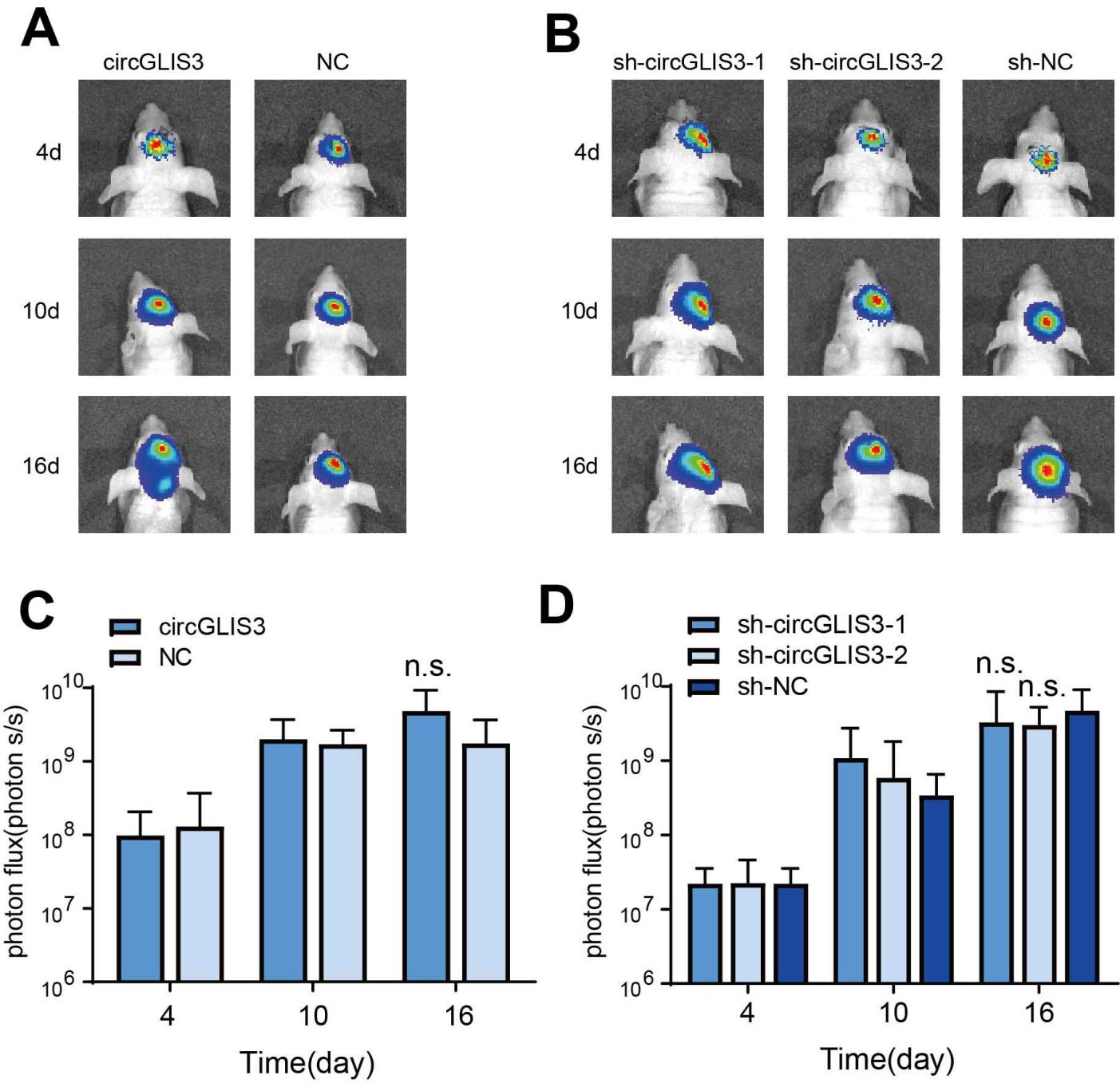


**Supplementary Figure 2.** **Up- or down regulating circGLIS3 in glioma intracranial xenograft model.** (**A**) and (**B**) IVIS lucfluorescence image of tumor bearing mice (n=5 for each group). Intracranial injected U87-MG cells were transfected with circGLIS3 plasmid or sh-circGLIS3 lentivirus with a corresponding control group. (**C**) and (**D**) The lucfluorescencee signal intensity of intracranial glioma (n=5 for each group).

**
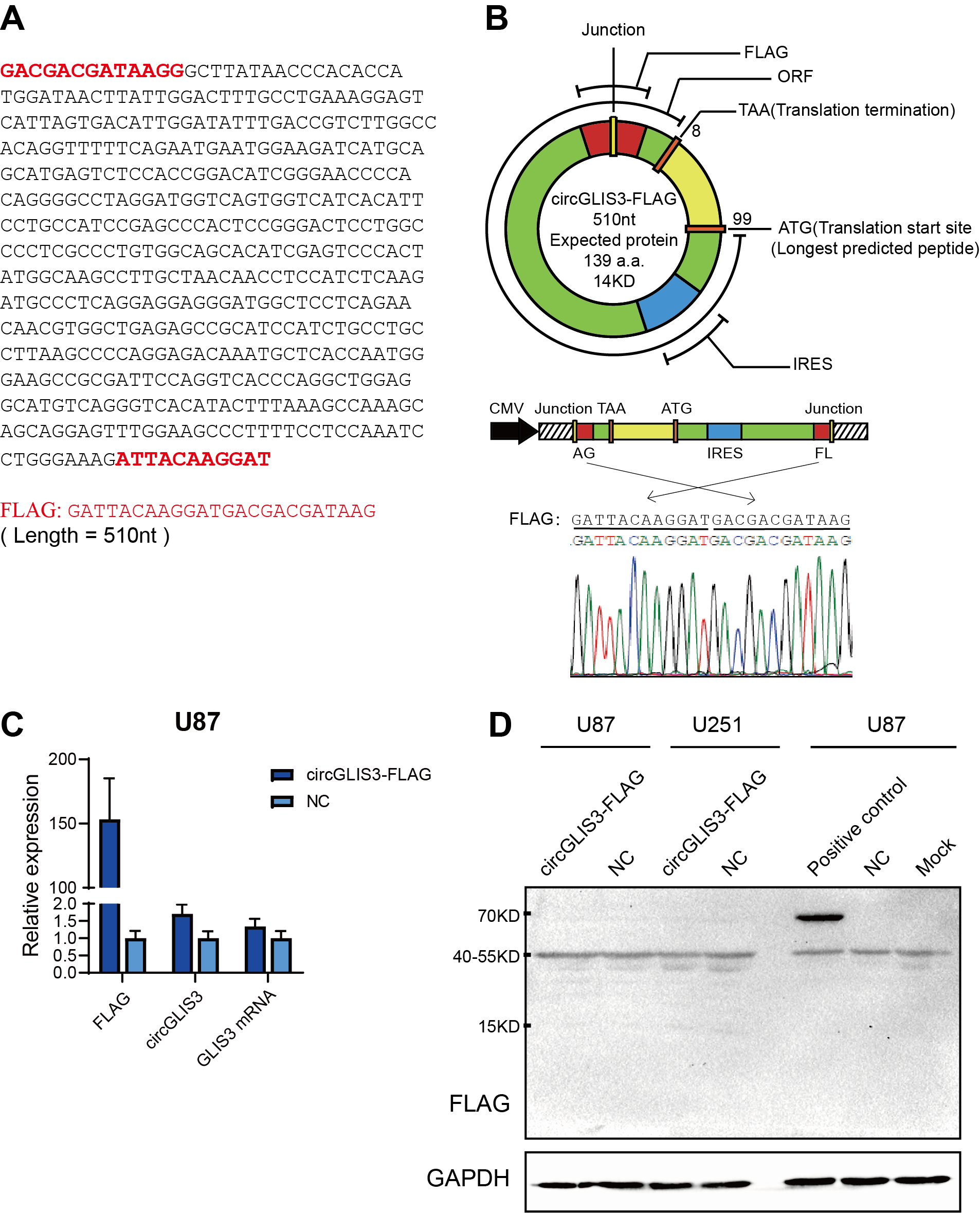
**

**Supplementary Figure 3. CircGLIS3 cannot be translated into peptide.** (**A**) Total sequence of designed FLAG-tagged circGLIS3. CircGLIS3 is flanked by back-spliced FLAG sequence. (**B**) Schematic diagram of FLAG-tagged circGLIS3(Upper) and Sanger sequencing of circGLIS3-FLAG qPCR divergent primers amplification products(Lower). Sequences can blast to circGLIS3-FLAG junction. (**C**) RT-PCR of U87 cells transfected by circGLIS3-FLAG or negative control plasmid. (**D**) Western blot of U87 and U251 cells transfected by circGLIS3-FLAG or negative control plasmid. Right shows positive control with a band around 70 KDa. The experiments were totally repeated for 3 times with 3 repetitions for each group.

## Supplementary Tables

| **Supplementary Table 1. characteristics of subjects with microarray and RT-PCR** | | | | |
| --- | --- | --- | --- | --- |
| ID# | Gender* | Age^ | Tissue | WHO grade |
| NBT1 | M | 44 | cortical brain | - |
| NBT2 | M | 5 | cortical brain | - |
| NBT3 | F | 18 | cortical brain | - |
| NBT6 | M | 11 | cortical brain | - |
| NBT7 | F | 24 | cortical brain | - |
| G1 | F | 33 | glioblastoma multiforme | IV |
| G2 | F | 54 | anaplastic astrocytoma | III |
| G3 | M | 48 | anaplastic oligodendroglioma | III |
| G4 | M | 31 | glioblastoma multiforme | IV |
| G5 | M | 23 | ganglioglioma | II |
| G6 | M | 23 | glioblastoma multiforme | IV |
| G7 | F | 67 | glioblastoma multiforme | IV |
| G8 | F | 54 | oligodendroglioma | II |
| G9 | M | 52 | glioblastoma multiforme | IV |
| G10 | M | 44 | glioblastoma multiforme | IV |
| G11 | M | 39 | anaplastic oligodendroglioma | III |
| G12 | M | 44 | oligodendroglioma | II |
| G16 | M | 72 | glioblastoma multiforme | IV |
| G17 | M | 7 | diffuse astrocytoma | II |
| G18 | F | 59 | anaplastic astrocytoma | III |
| G19 | F | 37 | anaplastic glioma | II |
| G20 | M | 61 | diffuse Astrocytoma | II |
| G21 | M | 22 | diffuse Astrocytoma | II |
| G22 | M | 61 | unique astrocytoma variants | II-III |
| G24 | M | 53 | glioblastoma multiforme | IV |
| G25 | M | 2 | gemistocytic astrocytoma | II |
| G26 | F | 31 | astrocytoma | II |
| G27 | M | 62 | glioblastoma multiforme | IV |
| G28 | F | 50 | glioblastoma multiforme | IV |
| G29 | M | 30 | ependymoma | II |
| G30 | M | 72 | anaplastic oligodendroglioma | III |
| G31 | F | 52 | oligodendroglioma | II |
| G33 | M | 33 | oligodendroglioma | II |
| #NBT = non-tumor brain tissue; G = glioma. | | | | |
| *M = Male; F = Female | | | | |
| ^Age at the time of autopsy | | | | |

| **Supplementary Table 2. designed primer information** | | |
| --- | --- | --- |
| **gene** | **forward** | **reverse** |
| circGLIS3 (divergent） | CCAGGAGACAAATGCTCACCA | GGTGTGGGTTATAAGCCTTTCC |
| circGLIS3 (convergent) | ATGGTCAGTGGTCATCACATTCC | GTTAGCAAGGCTTGCCATAGTG |
| GLIS3 | ACAACCCCTCCTCCCAGTTA | TGATGTGGTGAGGAGATGGA |
| circGLIS3-FLAG | GGGAAAGATTACAAGGATGACG | TCTGAAAAACCTGTGGCCAA |
| hsa_circ_0006370 | GCCCAACAAGTGTACGGTCC | TGAGAGCCGTGGTTGGAGAG |
| hsa_circ_0008494 | AGATTCATTTGGGTCTCAGGC | ATTGGACTGGATGGAGGCA |
| hsa_circ_0004058 | CCATTGGATAAGGATAAAGACACTC | CATTCAGAGGTAAGATAAGGTCGG |
| hsa_circ_0126678 | GTGGTGCCTCTCTGCTTATTGT | TTGTAGCACAGAACCCTGCAA |
| hsa_circ_0109937 | GGCAAAAGACCGTATGAATGTC | CCACACACACTCCAAGTCAGTATG |
| hsa_circ_0015275 | GATGGCACAACATACTTGAAGACC | GCCTCATCTACCAAGTCTCTCCAT |
| hsa_circ_0008827 | GGATATTCCAATGTTCTTTGTGGT | ATAGCAACTTGTGTGTCTGAAAGTG |
| GAPDH | ACAACTTTGGTATCGTGGAAGG | GCCATCACGCCACAGTTTC |

| **Supplementary Table 3. primary and secondary antibodies information.** | | | | |
| --- | --- | --- | --- | --- |
| **Antibody** | **Manufacture** | **Catalog No.** | **Application** | **RRID** |
| GLIS3 | abcam | ab126901 | WB 1:1000 | RRID:AB_11140555 |
| FLAG tag | sigma aldrich | F1804 | WB 1:1000 | RRID:AB_262044 |
| Ezrin | abcam | ab40839 | WB 1:1000, RIP 10μg | RRID:AB_732275 |
| p-Ezrin(T567) | abcam | ab76247 | WB 1:1000, RIP 10μg, IHC 1:100 | RRID:AB_1523584 |
| GAPDH | proteintech | 10494-1-AP | WB 1:10000 | RRID:AB_2263076 |
| ROCK2 | abcam | ab71598 | WB 1:1000, IF 1:100 | RRID:AB_1566688 |
| RhoA | abcam | ab54835 | WB 1:1000 | RRID:AB_945224 |
| RIOK1 | proteintech | 17222-1-AP | WB 1:1000, IF 1:100 | RRID:AB_2284990 |
| TSG101 | abcam | ab125011 | WB 1:1000 | RRID:AB_10974262 |
| CD9 | abcam | ab92726 | WB 1:1000 | RRID:AB_10561589 |
| ALIX | abcam | ab275377 | WB 1:1000 | - |
| goat anti rabbit-HRP | CST | 7074P2 | WB 1:20000 | RRID:AB_2099233 |
| goat anti mouse-HRP | Bioworld | BS12478 | WB 1:20000 | RRID:AB_2773727 |
| GAPDH-HRP | proteintech | HRP-60004 | WB 1:10000 | RRID:AB_2737588 |
| Alexa Fluor®488 conjugated antibody | abcam | ab150113 | IF 1:200 | RRID:AB_2576208 |
| Alexa Fluor®594 conjugated antibody | abcam | ab150080 | IF 1:200 | RRID:AB_2650602 |

| **Supplementary Table 4. Top 10 up expressed circRNAs among NBT, LGG and HGG** | | | | | | | |
| --- | --- | --- | --- | --- | --- | --- | --- |
| LGG vs NBT | | | | | | | |
| **Alias** | **source** | **P-value** | **FC (abs)** | **chrom** | **txStart** | **txEnd** | **GeneSymbol** |
| hsa_circ_0091419 | circBase | 0.016 | 18.419 | chrX | 118920468 | 118923974 | RPL39 |
| hsa_circ_0082326 | circBase | 0.002 | 16.792 | chr7 | 129665997 | 129679387 | ZC3HC1 |
| hsa_circRNA_406281 | 25070500 | 0.007 | 13.787 | chr3 | 52723939 | 52724314 | GNL3 |
| hsa_circ_0040994 | circBase | 0.003 | 13.270 | chr16 | 89807211 | 89815175 | FANCA |
| hsa_circ_0000463 | circBase | 0.004 | 12.842 | chr12 | 132609079 | 132609271 | EP400NL |
| hsa_circ_0044097 | circBase | 0.001 | 12.275 | chr17 | 42929776 | 42932372 | EFTUD2 |
| hsa_circ_0001568 | circBase | 0.019 | 11.999 | chr6 | 349113 | 349256 | DUSP22 |
| hsa_circRNA_406157 | 25070500 | 0.016 | 11.990 | chr21 | 45302384 | 45323900 | AGPAT3 |
| hsa_circ_0001275 | circBase | 0.004 | 11.730 | chr3 | 17059499 | 17059748 | PLCL2 |
| hsa_circ_0051799 | circBase | 0.002 | 11.617 | chr19 | 49458943 | 49464519 | BAX |
|  |  |  |  |  |  |  |  |
| HGG vs NBT | | | | | | | |
| **Alias** | **source** | **P-value** | **FC (abs)** | **chrom** | **txStart** | **txEnd** | **GeneSymbol** |
| hsa_circ_0040994 | circBase | 0.001 | 11.982 | chr16 | 89807211 | 89815175 | FANCA |
| hsa_circ_0000463 | circBase | 0.004 | 11.147 | chr12 | 132609079 | 132609271 | EP400NL |
| hsa_circ_0044097 | circBase | 0.001 | 10.855 | chr17 | 42929776 | 42932372 | EFTUD2 |
| hsa_circ_0028912 | circBase | 0.000 | 10.680 | chr12 | 121004627 | 121009094 | RNF10 |
| hsa_circ_0002082 | circBase | 0.001 | 9.683 | chr11 | 65271199 | 65272066 | MALAT1 |
| hsa_circRNA_405324 | 25070500 | 0.000 | 9.633 | chr15 | 43008874 | 43011764 | STARD9 |
| hsa_circ_0074660 | circBase | 0.000 | 9.351 | chr5 | 151122382 | 151138210 | ATOX1 |
| hsa_circ_0001275 | circBase | 0.005 | 9.107 | chr3 | 17059499 | 17059748 | PLCL2 |
| hsa_circRNA_406281 | 25070500 | 0.019 | 9.007 | chr3 | 52723939 | 52724314 | GNL3 |
| hsa_circ_0037274 | circBase | 0.000 | 8.990 | chr16 | 981641 | 1004666 | LMF1 |
| hsa_circ_0002874 (top 32) | circBase | 0.009 | 5.302 | chr9 | 4286037 | 4286523 | GLIS3 |
|  |  |  |  |  |  |  |  |
| HGG vs LGG | | | | | | | |
| **Alias** | **source** | **P-value** | **FC (abs)** | **chrom** | **txStart** | **txEnd** | **GeneSymbol** |
| hsa_circ_0002874 | circBase | 0.027 | 4.268 | chr9 | 4286037 | 4286523 | GLIS3 |
| hsa_circ_0006370 | circBase | 0.019 | 3.306 | chr9 | 4117767 | 4118881 | GLIS3 |
| hsa_circ_0088194 | circBase | 0.016 | 2.874 | chr9 | 117798376 | 117808961 | TNC |
| hsa_circ_0088200 | circBase | 0.010 | 2.579 | chr9 | 117819431 | 117819704 | TNC |
| hsa_circ_0004822 | circBase | 0.044 | 2.525 | chr9 | 4286037 | 4286435 | GLIS3 |
| hsa_circ_0052154 | circBase | 0.041 | 2.500 | chr19 | 54711265 | 54711515 | RPS9 |
| hsa_circRNA_406076 | 25070500 | 0.029 | 2.411 | chr2 | 243056789 | 243081039 | LOC728323 |
| hsa_circRNA_407145 | 25070500 | 0.005 | 2.396 | chr9 | 3932359 | 3937189 | GLIS3 |
| hsa_circ_0067344 | circBase | 0.042 | 2.157 | chr3 | 130851588 | 130852800 | NEK11 |
| hsa_circ_0079672 | circBase | 0.047 | 2.107 | chr7 | 28527792 | 28547355 | CREB5 |
